# The possible renoprotective effect of denatonium benzoate in a rat model of type 2 diabetes: role of Krüppel-like factor 6 (KLF6)

**DOI:** 10.1007/s00210-025-04704-9

**Published:** 2025-11-05

**Authors:** Hend S. Zakaria, Ola S. El-Fetiany, Walaa Omar, Walaa N. Roushdy

**Affiliations:** 1 Clinical Pharmacology Department, Faculty of Medicine, Alexandria University, Alexandria, Egypt; 2 Medical Physiology Department, Faculty of Medicine, Alexandria University, Alexandria, Egypt; 3 Histology and Cell Biology Department, Faculty of Medicine, Alexandria University, Alexandria, Egypt; 4 Medical Biochemistry Department, Faculty of Medicine, Alexandria University, Alexandria, Egypt

**Keywords:** Bitter taste-sensing type 2 receptors (TAS2Rs), Krüppel-like factor (KLF6), Diabetes mellitus

## Abstract

Bitter taste-sensing type 2 receptors (TAS2Rs) were first discovered in the oral cavity, but their detection in extra-oral tissues like gastrointestinal tract and kidney leads to modulation of multiple (patho) physiological processes. Krüppel-like factor (KLF6) is a novel regulator of β-cell adaptation to metabolic stress and involved in multiple processes in vascular system. This research aimed to assess the possible metabolic and renal protective effect of TAS2R agonist denatonium benzoate and the antidiabetic drug sitagliptin in diabetic rats. Forty male Sprague Dawley rats were included and divided into two main groups: normal control group and fructose-fed streptozotocin-induced diabetic group. Diabetic group was subdivided into four subgroups untreated: sitagliptin, denatonium, and sitagliptin + denatonium-treated groups**.** All treated groups showed improvement of glycemic and lipid profiles and UACR with enhancement of renal KLF6 expression and improved histological architecture of diabetic kidney. The combination group showed the best results with most renal corpuscles returning to normal and biochemical values close to normal control. Compared to the diabetic control group, the combination group showed significantly lower fasting blood glucose, HbA1C, UACR, TGs, and LDL-C values (≈3.44, 2.1, 3.5, 1.6, and 1.5-fold reduction, respectively), and significant elevation of serum GLP, HDL-C, and renal KLF6 expression (≈2.1, 2.0, and 3.7-fold increase, respectively). Significant correlations were detected between KLF6 expression and various parameters in all rats of the experiment. This study concluded the promising role of denatonium benzoate and its combination with sitagliptin in the management of diabetes and its renal complications.

**Supplementary Information:**

The online version contains supplementary material available at 10.1007/s00210-025-04704-9.

## Introduction

TAS2Rs, or bitter taste-sensing type 2 receptors, are a subclass of G-protein coupled receptors (GPCRs) of family A. Taste bud cells in the oral cavity were the first site to discover bitter taste receptors. They serve as sensors of bitter, toxic substances to stop them from being consumed (Liman et al. 2014). After more investigation, these receptors were found in a diversity of extraoral tissues and are thought to be a key modulator of various (patho)physiological processes in addition to the perception of bitter chemicals (Tuzim and Korolczuk 2021) .

The stimulation of TAS2Rs on the surface of gut enteroendocrine L cells in the gastrointestinal tract causes an increase in intracellular Ca2 + and the release of the incretin hormone glucagon-like peptide-1 (GLP-1), which is a crucial regulator of insulin production and biosynthesis (Dotson et al. 2008). It has been proposed that activation of TAS2Rs may have a positive effect on glucose homeostasis and may be a new target in metabolic disorder treatment, including type 2 diabetes mellitus (T2D), because bitter compounds have the ability to stimulate intestinal hormone secretion, especially incretins (Dotson et al. 2010).

Diabetic kidney disease (DKD) is the principal cause of kidney failure all over the world and its prevalence did not decline over the past 30 years (Koye et al. 2018). Hyperglycemia is the chief etiological factor for the development of diabetic kidney disease. Numerous pathophysiological abnormalities, such as oxidative stress, abnormal tubule-glomerular feedback, renal podocyte damage, inflammatory states, endothelial dysfunction, and progressive glomerulosclerosis, arise once hyperglycemia is established. These abnormalities ultimately result in the pathology of diabetic nephropathy (DN) (DeFronzo et al. 2021).

Numerous physiological processes, including differentiation, proliferation, metabolism, oxidative stress, and inflammation, are impacted by the zinc finger DNA-binding proteins that make up the Krüppel-like factor (KLF) family. One of the KLF family’s essential members, KLF6 has been revealed to be a new regulator of β-cell adaptation to metabolic stress. Angiogenesis, vascular repair, and remodeling following vascular injury are all regulated by KLF6 in the vascular system (Pollak et al. 2018). Moreover, Krüppel-like factors (KLFs) were found to have a crucial role in glomerular biology. Nevertheless, further research is essential to determine the precise mechanism by which KLF6 is controlled in diabetes situations. It was shown that KLF6 plays a crucial role in moderating mitochondrial damage in diabetic podocytes by controlling the production of cytochrome c oxidase (COX), which prevents cytochrome c from being released and apoptosis from being triggered in response to cell stress (Mallipattu et al. 2015).

By making podocytes more vulnerable to mitochondrial malfunction and triggering the intrinsic apoptotic pathway, Klf6 knockdown accelerates up DKD (Horne et al. 2018). These effects suggest that KlF6 overexpression may be a therapeutic or preventive strategy against T2D development, and its vascular complications (Dumayne et al. 2020).

Sitagliptin is an antidiabetic medication of the gliptin class. It acts via inhibition of dipeptidyl peptidase-4 (DPP-4), an enzyme which acts to breakdown and inactivate GLP-1. It offers renoprotective effects through antioxidant, anti-inflammatory, and antiapoptotic mechanisms (Mohamed et al. 2022).

Denatonium benzoate (DB) is one of the TAS2R agonists (Salvestrini et al. 2020). It may exert antidiabetic activity and positively impacts blood glucose levels possibly through activation of TAS2Rs and increasing GLP-1 secretion (Kim et al. 2014). This research aimed to assess the possible metabolic and renal protective effect of denatonium benzoate alone and in combination with sitagliptin in fructose-fed streptozotocin-induced diabetic rats focusing on the modulation of Krüppel-like factor 6 (KLF6).

## Material and methods: Fig. 1

**Fig. 1:**
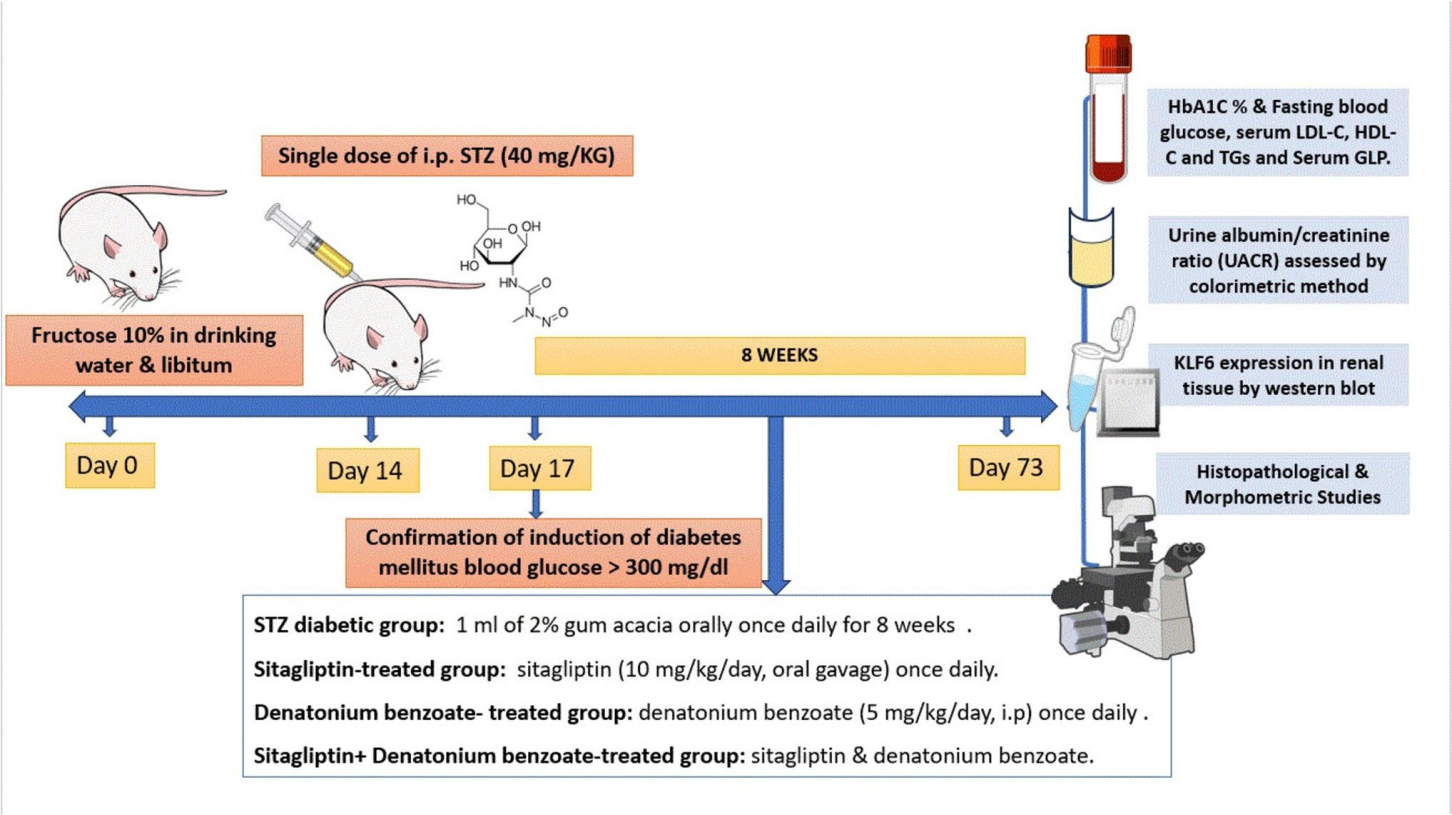
Flow chart for the experimental design of the study. T2D was induced by adding fructose 10% to drinking water ad libitum, then the injection of single dose of streptozotocin (STZ) (40 mg/kg) at the end of the second week. Rats were treated for 8 weeks, then sacrificed for biochemical and histological assessment of the kidney. Images provided by the NIH BioArt source https://bioart.niaid.nih.gov/bioart/283, https://bioart.niaid.nih.gov/bioart/506, https://bioart.niaid.nih.gov/bioart/143, https://bioart.niaid.nih.gov/bioart/52, https://bioart.niaid.nih.gov/bioart/503 were used in this chart

### Animals

Forty male Sprague Dawley rats whose weight ranged between 150 and 200 g were used for this study. Animals were housed in the Medical Physiology department animal house, Alexandria Faculty of Medicine, Egypt. The rats were kept in animal cages with four rats per cage, at a temperature of 23–27 °C, and a 12/12-h light/dark cycle with free access to water and food. Animals were acclimatized to housing conditions for 1 week before starting the study. The study protocol has been approved by the Research Ethics Committee of the Alexandria Faculty of Medicine (Ethical registration NO: 0306098, IRB NO: 00012098-FWA NO: 00018699), and all methods were carried out in accordance with the Animal Research: Reporting of In Vivo Experiments (ARRIVE) guidelines (Percie du Sert et al. 2020) .

### Drugs and chemicals

Fructose (CAS 57–48-7), streptozotocin (CAS 18883–66-4), gum acacia (CAS 9000–01–5), sitagliptin (CAS 486460–32-6), denatonium benzoate (CAS 3734–33-6), ketamine (CAS 1867–66-9), and xylazine (CAS 23076–35-9) were acquired from Sigma-Aldrich (Saint Louis, USA). Citrate buffer solution (prepared from citric acid monohydrate, CAS 5949–29-1, and sodium citrate dihydrate, CAS 6132–04-3) was obtained from Arab Laboratory Scientific Equipment (Ras Al Khaimah, UAE).

### Study design and experimental grouping

Randomly, animals were divided into two main groups: **normal control group** (*n* = 8) in which animals received normal drinking water throughout the study period. At the end of the second week, a single intraperitonial (i.p) injection of 1 ml 0.1 molar citrate buffer was given to rats in this group, then 1 ml of gum acacia (2%) was administered orally once daily for 8 weeks. The second group was **type 2 diabetic group** (**fructose-fed streptozotocin-induced diabetic rats**) (*n* = 32) in which T2D was induced by adding fructose 10% to drinking water and libitum, then a single i.p injection with streptozotocin (STZ) (40 mg/kg) dissolved in citrate buffer (pH 4.4) was given to the animals at the end of the second week. After 72 h of the STZ injection, rats with non-fasting blood glucose levels more than 300 mg/dl were diagnosed as diabetic and enrolled in the study (Wilson and Islam 2012). The diabetic group was further subdivided into four subgroups (eight rats each): **diabetic control group**: in which 1 ml of 2% gum acacia was given orally once daily for 8 weeks following the induction of DM; **sitagliptin-treated diabetic group**: in which rats received sitagliptin (10 mg/kg/day, oral gavage) suspended in 1 ml of gum acacia (2%) once daily for 8 weeks following the induction of DM (Mohamed et al. 2022); **denatonium benzoate-treated diabetic group**: rats received denatonium benzoate (5 mg/kg/day, i.p) suspended in 1 ml of gum acacia (2%) once daily for 8 weeks following the induction of DM (Kim et al. 2014); **sitagliptin + denatonium benzoate-treated diabetic group**: rats received sitagliptin and denatonium benzoate as mentioned in the treated groups.

### Study termination and sample collection

After 8 weeks, the rats were anaesthetized using intraperitoneal injection containing combination of ketamine (50 mg) and xylazine (5 mg/kg). They were sacrificed, and different samples were collected as follows:

- **Urine samples**: Urine samples were collected 24 h before scarification. This was performed by housing each rat in a special metabolic cage with perforated platform to collect the urine samples starting from 8 a.m. to 8 a.m. next day. Urine albumin/creatinine ratio (UACR) was assessed using colorimetric method (Assadi 2002).
- **Blood samples**: Following animal scarification, blood samples were obtained from the abdominal aorta and utilized to assess glycemic and lipid profiles by measuring fasting blood glucose (fasting for 12 h), glycosylated hemoglobin (HbA1C%), serum GLP, TGs, LDL-C, and HDL-C.
- **Tissue samples**: Both kidneys were isolated in a sterile setting. One kidney was allocated for assessment of KLF6 by western blot analysis, while the other one was prepared for histopathological examination.

### Biochemical analysis

#### Measurement of urine albumin/creatinine ratio (UACR)

Urinary albumin level was measured using commercially available Rat ELISA kit obtained from (Biorbyt, NC, USA, Catalog No. orb2810077) following the manufacturer’s instructions. Samples were assayed in duplicate, and absorbance was measured at 450 nm. Albumin levels were expressed as µg/ml.

Urinary creatinine level was measured using colorimetric kit purchased from (Cell Biolabs, San Diego, USA, Catalog No. STA-378). Samples and standards were incubated for 30 min with a reaction reagent which changes color from yellow to bright orange upon reacting with creatinine, forming the creatinine-picrate complex. The O.D values were read at 490 nm. Samples were compared to the known concentration of creatinine standard, and results were presented as mg/dl.

For calculation of UACR, both values were converted into the same volume unit (per ml). Albumin (µg/ml) was divided by creatinine (mg/ml), and the ratio was expressed as µg albumin per mg creatinine (µg/mg).

#### Assessment of glycemic profile

Serum levels of glucose and HbA1C% were determined using commercially available colorimetric kits obtained from (Spectrum diagnostics, Cairo, Egypt, Catalog No. 250001 and 255,002 respectively).

Glucagon-like peptide (GLP) serum level was measured using Rat ELISA assay kit from (FineTest Co., Wuhan, China, Catalog No. ER0996). The samples were tested twice on a microplate at 450 nm, and results were expressed as pg/ml.

#### Assessment of lipid profile

Colorimetric assay kits were provided by Linear Chemicals S.L.U (Barcelona, Spain) for measuring serum levels of TGs (Catalog No. 1155010), LDL-C (Catalog No. 1133105), and HDL-C (Catalog No. 1133010).

### Detection of Krüppel-like factor 6 (KLF6) expression in renal tissue by western blot

Western blot was used to measure the expression of Krüppel-like factor 6 (KLF6) protein in renal tissues. For homogenization of renal tissue, radioimmunoprecipitation (RIPA) lysis buffer (Catalog No. AR0105, Bosterbio, CA, USA) supplemented with 1% (v/v) protease inhibitor cocktail (Catalog No. AR1182, Bosterbio, CA, USA) and 50 mM Tris–HCl (pH 8.0) was used to prepare the tissue lysate. Lowry method was used to measure the total protein concentration of the lysates (Waterborg 2009). The lysates were stored at − 80 °C until analysis.

Equal amounts of protein (30 µg per lane in 20 µl Laemmli sample buffer “Catalog No. S3401, Sigma-Aldrich, Saint Louis, USA”) were separated by electrophoresis on 10% sodium dodecyl sulfate–polyacrylamide gels (SDS-PAGE). The proteins were then transferred onto 0.45-µm nitrocellulosemembranes (Thermo Fisher Scientific, Fermentas, USA) by electroblotting using transfer buffer (25 mM Tris, 192 mM glycine, 20% methanol) at 100 V for 90 min.

Membranes were blocked by incubation with 5% non-fat milk in Tris-buffered saline with Tween 20 (TBST; 20 mM Tris–HCl, pH 7.5, 150 mM NaCl, 0.1% Tween 20) for 60 min at room temperature, followed by three washes (10 min each) with TBST (10 ml per membrane). Primary antibody incubation was performed overnight at 4 °C using Anti-KLF6 polyclonal antibody (1:1000, Catalog No. YPA2473, Biospes, Chongqing, China) and Anti-β-Actin (1:5000, Catalog No. PA1-16,889, Thermo Fisher Scientific, Fermentas, USA), both diluted in 10 ml of TBST containing 1% bovine serum albumin (BSA).

After washing three times with TBST, membranes were incubated with anti-rabbit IgG-HRP-conjugated secondary antibody (1:10,000, Catalog No. 31460, Thermo Fisher Scientific, Fermentas, USA) for 60 min at room temperature. Membranes were again washed three times with TBST, and bands were visualized using DAB Horseradish Peroxidase Chromogenic Kit (Catalog No. BWR1068, Biospes, Chongqing, China). A working solution was prepared according to the manufacturer’s instructions, and 10 ml was applied per membrane. The reaction was stopped by rinsing membranes with distilled water after 3–5 min of color development.

Relative band densities were quantified using ImageJ software (http://imagej.nih.gov/ij/), and protein expression was normalized against β-actin and expressed as fold change relative to control.

### Histopathology and morphometric studies

One kidney was cut into small specimens and fixed in 10% formol saline followed by processing by standard procedures and then the specimens were embedded in paraffin wax, and cut into small sections of 5-μm thickness. The specimens were stained by hematoxylin and eosin (H&E) stain for examination of structural changes. Sections were inspected using an Olympus LM (Olympus BX41) fitted with spot digital camera (Olympus DP20) at Center of Excellence for Research in Regenerative Medicine and Applications (**CERRMA**), Faculty of Medicine, Alexandria University (Kaur et al. 2017).

#### Quantitative morphometric analysis of renal cortices

MATLAB software (image J, THE MATHWORKS, Inc., USA) was used to view and record images taken using an Olympus microscope supplied with a Spot digital camera. A 10 × object lens was used to take pictures of a randomly selected cross section taken from each group of rats' renal corpuscles. Each group’s ten randomly chosen glomeruli were assessed. In ten glomeruli of each group, ten values for intracorpuscular nuclei count were calculated. Each group was evaluated for thirty mesangial expansion, entire renal corpuscular volume, and Bowman’s space values (three measurements in 10 glomeruli). Based on the mean number of pixels, the values for Bowman’s space, mesangial expansion, intracorpuscular nucleus cell count, and entire renal corpuscular volume were calculated. Quantitative data for each group was presented as mean ± standard deviation (SD) (Olivetti et al. 1980).

Kidney tissue damage scoring was assessed using the morphometric data according to the following semiquantitative scores: glomerular hypertrophy (0–3), mesangial expansion (0–3), intracorpuscular cellularity (0–3), and Bowman’s space (0–3) (Roufosse et al. 2018) and (Venkatareddy et al. 2014).

### Statistical analysis

Version 20.0 of the IBM SPSS software package was used to examine the biochemical and morphometric data (IBM Corp., Armonk, NY). The normality of distribution was tested by the Kolmogorov–Smirnov test. For normally distributed data, one-way analysis of variance (ANOVA) followed by Tukey’s multiple comparison post hoc test was performed to compare group means. *p* < 0.05 was considered statistically significant. Results were expressed as mean ± standard deviation (SD). Pearson’s correlation coefficient was used to evaluate the relationship between two normally distributed quantitative variables.

## Results

### Biochemical analysis: Table 1 and Figs. 2, 3, 4, and 5

**Table 1:** Comparison between the different studied groups according to different parameters

	Normal control	Diabetic control	Sitagliptin	Denatonium	Sitagliptin+dentonium	*F*	*p*
FBG (mg/dl)							
Min.–Max.	70.0–95.0	290.0–350.0	90.0–125.0	119.0–160.0	80.0–100.0	413.75^**^	<0.001
Mean ± SD.	78.50 ± 7.86	307.5^*^± 19.64	108.5^*#^ ± 13.59	131.8^*#@^± 13.17	89.38^#@$^± 7.11		
HbA1C (mmol/mol (%)							
Min.–Max.	3.0–4.20	6.0–8.50	3.90–5.70	5.50–7.50	3.0–4.20	61.578^**^	<0.001
Mean ± SD.	3.75 ± 0.40	7.40^*^ ± 0.82	4.66^*#^ ± 0.67	6.46^*#@^ ± 0.67	3.53^#@$^ ± 0.40		
HDL-C (mg/dl)							
Min.–Max.	40.0–65.0	19.0–27.0	30.0–50.0	30.0–40.0	30.0–60.0	22.008^**^	<0.001
Mean ± SD.	51.87 ± 8.61	22.75^*^± 3.15	44.50^#^± 6.72	33.38^*#@^ ± 4.03	46.13^#$^ ± 9.99		
(LDL-C) (mg/dl)							
Min.–Max.	28.0–40.0	80.0–105.0	59.0–70.0	65.0–95.0	49.0–69.0	64.680^**^	<0.001
Mean ± SD.	32.0 ± 3.82	90.25^*^ ± 10.07	64.0^*#^ ± 4.34	76.63^*#@^ ± 11.01	58.63^#@$^ ± 6.16		
(TGs) (mg/dl)							
Min.–Max.	65.0–80.0	200.0–240.0	120.0–180.0	180.0–200.0	115.0–150.0	142.70^**^	<0.001
Mean ± SD.	73.0 ± 6.05	210.6^*^± 13.48	139.4^*#^ ± 18.79	191.0^*#@^ ± 9.68	131.3^*#$^± 12.57		
(UACR) (μg/mg)							
Min.–Max.	13.0–26.0	140.0–280.0	70.0–110.0	99.0–170.0	30.0–75.0	54.76^**^	<0.001
Mean ± SD.	18.75 ± 4.74	195.6^*^ ± 48.51	95.63^*#^ ± 14.0	139.9^*#@^ ± 26.41	56.25^#@$^ ± 15.75		
Serum GLP (pg/ml)							
Min.–Max.	12.0–13.0	5.0–6.0	7.0–10.0	6.50–9.50	10.0–12.90	101.63^**^	<0.001
Mean ± SD.	12.53 ± 0.40	5.63^*^ ± 0.33	8.56^*#^± 0.90	8.13^*#^ ± 1.06	11.83^#@$^± 0.98		
Klf-6 (pg/ml)							
Min.–Max.	3.20–4.70	0.60–0.97	1.90–4.0	1.0–2.50	1.70–4.0	30.35^**^	<0.001
Mean ± SD.	3.95 ± 0.57	0.80^*^± 0.12	2.90^*#^± 0.81	1.81^*#@^ ± 0.44	2.96^*#$^ ± 0.86		

Eight replicas in each group

*SD*, standard deviation

*F*, *F* for one-way ANOVA test, pairwise comparison bet. each two groups was done using post hoc test (Tukey)

*p*, *p* value for comparing between the studied groups

**Statistically significant at *p* ≤ 0.05

Data are expressed as the mean±SD of eight rats per group. *p<0.05 significant vs. normal control, #*p*<0.05 significant vs. diabetic control, @*p*<0.05 significant vs. sitagliptin, $*p*<0.05 significant vs. denatonium

**Fig. 2:**
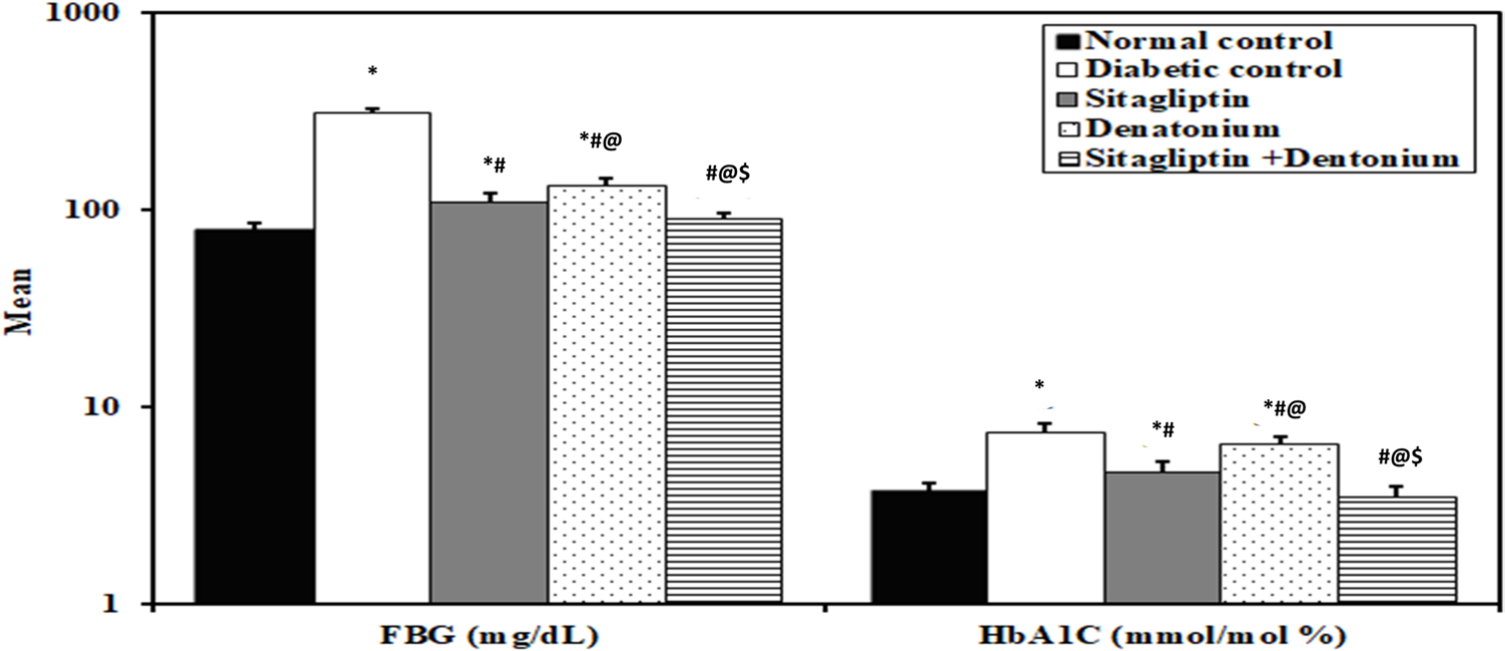
Effect of 8-week treatment with sitagliptin and denatonium alone or in combinations on FBG (mg/dl) and HbA1C (mmol/mol) (%) in fructose-fed streptozotocin-induced diabetic rats. Data are expressed as the mean±SD of eight rats per group. *p<0.05 significant vs. normal control, #*p*<0.05 significant vs. diabetic control, @*p*<0.05 significant vs. sitagliptin, $*p*<0.05 significant vs. denatonium

**Fig. 3:**
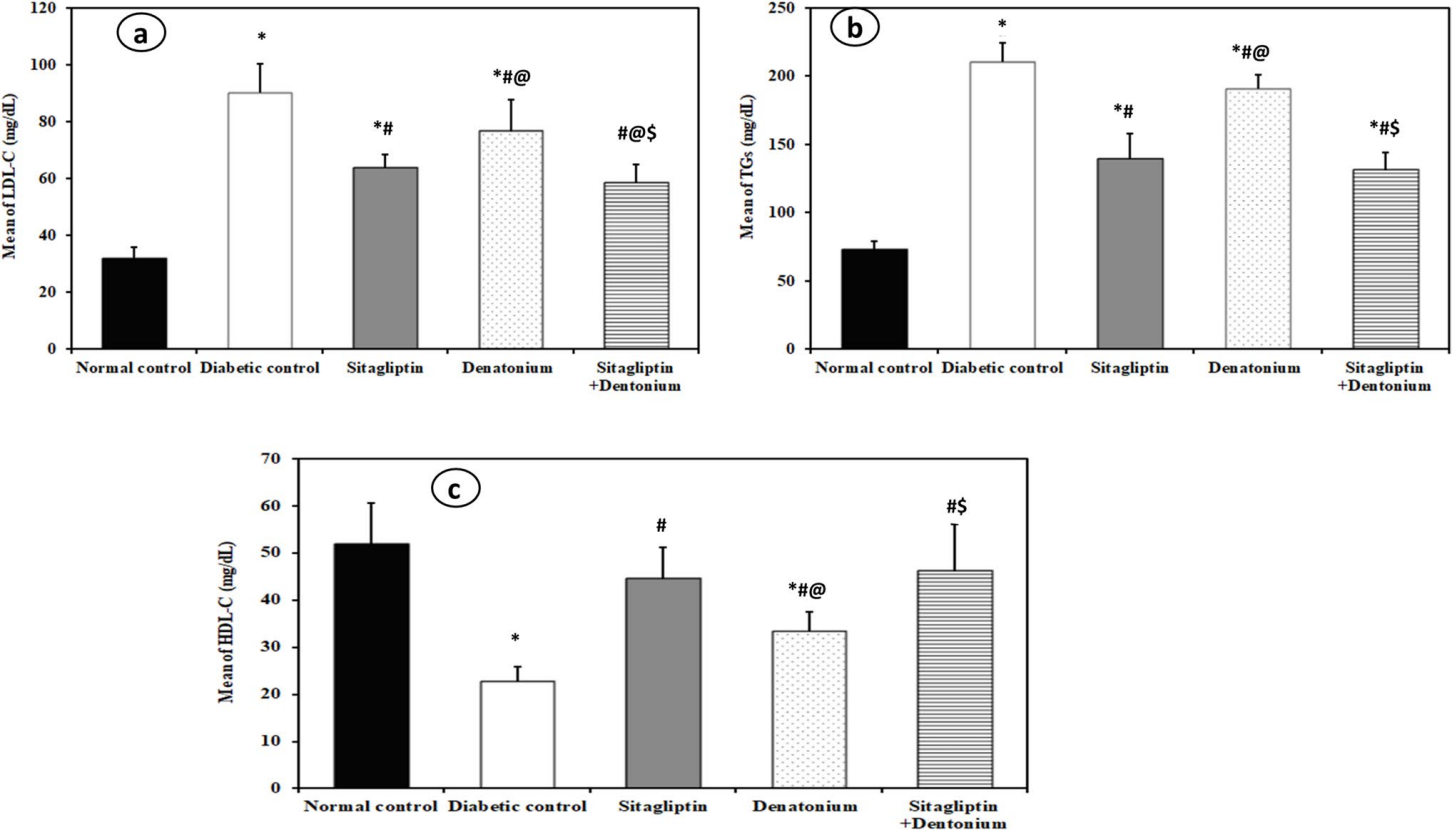
Effect of 8-week treatment with sitagliptin and denatonium alone or in combinations on LDL-C (mg/dl) in **a**, TGs (mg/dl) in **b**, and HDL-C (mg/dl) in **c** in fructose-fed streptozotocin-induced diabetic rats. Data are expressed as the mean±SD of eight rats per group. *p<0.05 significant vs. normal control, #*p*<0.05 significant vs. diabetic control, @*p*<0.05 significant vs. sitagliptin, $*p*<0.05 significant vs. denatonium

**Fig. 4:**
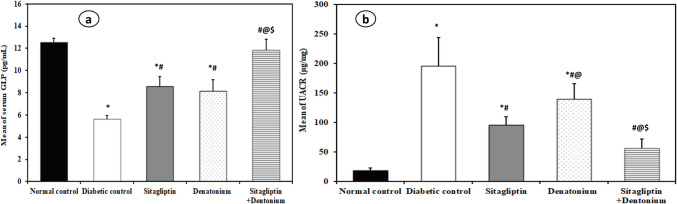
Effect of 8-week treatment with sitagliptin and denatonium alone or in combinations on serum GLP (pg/ml) in **a** and UACR (μg/mg) in **b** and in fructose-fed streptozotocin-induced diabetic rats. Data are expressed as the mean±SD of eight rats per group. *p<0.05 significant vs. normal control, #*p*<0.05 significant vs. diabetic control, @*p*<0.05 significant vs. sitagliptin, $*p*<0.05 significant vs. denatonium

**Fig. 5:**
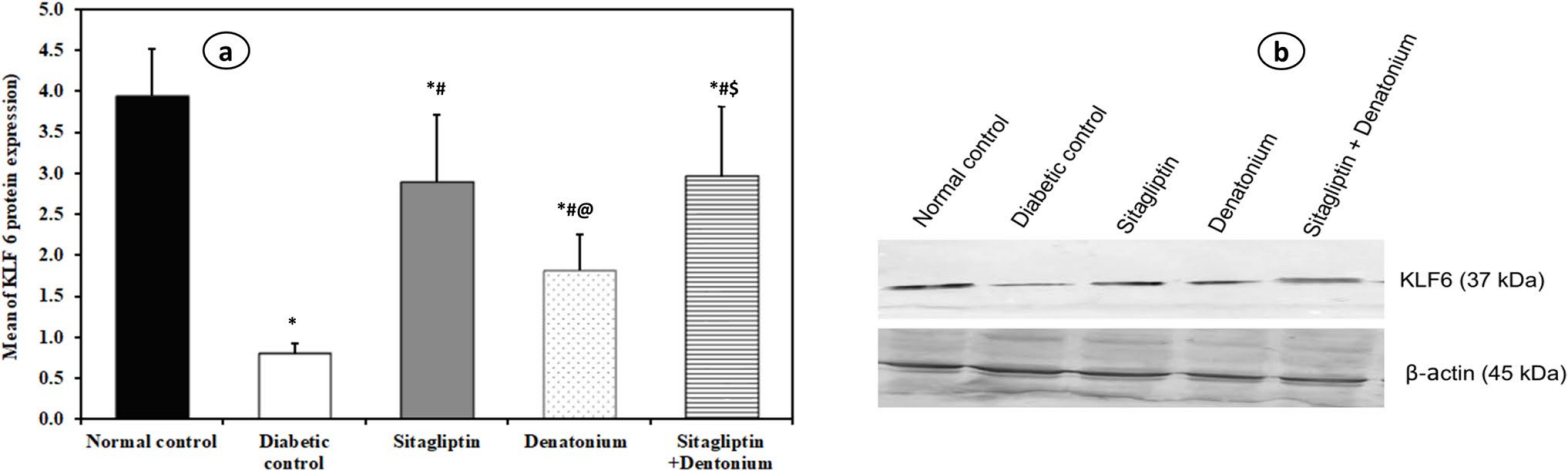
Effect of 8-week treatment with sitagliptin and denatonium alone or in combinations on KLF6 protien expression in **a** fructose-fed streptozotocin-induced diabetic rats and representative immunoblot for the expression of KLF6 in the kidney in **b**. Data are expressed as the mean±SD of eight rats per group. *p<0.05 significant vs. normal control, #*p*<0.05 significant vs. diabetic control, @*p*<0.05 significant vs. sitagliptin, $*p*<0.05 significant vs. denatonium

#### Diabetic control rats exhibited deterioration in all studied parameters

In the diabetic control rats, the levels of FBG (307.5 ± 19.64 mg/dl), HbA1C% (7.40 ± 0.82%), TGs (210.6 ± 13.48 mg/dl), and LDL-C (90.25 ± 10.07 mg/dl) were statistically significantly higher than the normal control group (78.50 ± 7.86 mg/dl, 3.75 ± 0.40%, 73.0 ± 6.05 mg/dl, 32.0 ± 3.82 mg/dl respectively), while they showed a significant decrease in the level of HDL-C (22.75 ± 3.15 mg/dl) as compared to the normal control group (51.87 ± 8.61 mg/dl). Moreover, there was a statistically significant increase in UACR in diabetic control group (195.6 ± 48.51 μg/mg) as compared to the normal control group (18.75 ± 4.74 μg/mg), while serum GLP level and renal KLF6 expression were significantly lower in diabetic control group (5.63 ± 0.33 pg/ml and 0.80 ± 0.12 pg/ml respectively) than the normal control group (12.53 ± 0.40 pg/ml and 3.95 ± 0.57 pg/ml respectively).

#### Administration of sitagliptin and denatonium benzoate improved glycemic profile (FBG & HbA1C%) in treated rats

The levels of FBG and HbA1C% in sitagliptin-treated group (108.5 ± 13.59 mg/dl and 4.66 ± 0.67%) and denatonium-treated group (131.8 ± 13.17 mg/dl and 6.46 ± 0.67%) showed a statistically significant decline than the diabetic control group (307.5 ± 19.64 mg/dl and 7.40 ± 0.82%) with a significant difference between the two groups. The sitagliptin + denatonium benzoate combination group showed statistically significant lower levels of FBG (89.38 ± 7.11 mg/dl) and HbA1C% (3.53 ± 0.40%) than the diabetic control group and the single drug groups.

#### Improved glycemic profile was associated with increased serum GLP in treated animals

The serum GLP level in both sitagliptin and denatonium-treated groups (8.56 ± 0.90 and 8.13 ± 1.06 pg/ml respectively) was statistically significant higher than the diabetic control group (5.63 ± 0.33 pg/ml) without significant difference between the two groups. When compared to the diabetic control and single drug groups, the sitagliptin + denatonium benzoate combination group showed a statistically significant higher serum GLP level (11.83 ± 0.98 pg/ml).

#### Sitagliptin and denatonium treatment enhanced lipid profile (TGs, LDL-C, HDL-C) in treated groups

Both sitagliptin and denatonium-treated groups demonstrated significantly lower TG levels (139.4 ± 18.79 and 191.0 ± 9.68 mg/dl respectively) and LDL-C levels (64.0 ± 4.34 and 76.63 ± 11.01 mg/dl respectively) with a significant higher HDL-C level (44.50 ± 6.72 and 33.38 ± 4.03 mg/dl respectively) when compared to the diabetic control group (210.6 ± 13.48, 90.25 ± 10.07, and 22.75 ± 3.15 mg/dl respectively), with significant differences between the two treated groups.

The sitagliptin + denatonium benzoate combination group exhibited a statistically significant reduction in the levels of TGs (131.3 ± 12.57 mg/dl) and LDL-C (58.63 ± 6.16 mg/dl) and a significant increase in HDL-C level (46.13 ± 9.99 mg/dl) compared to the diabetic control group and the denatonium-treated group.

#### Sitagliptin and denatonium treatment resulted in an improvement in UACR

Sitagliptin and denatonium-treated groups exhibited a statistically significant decrease in UACR (95.63 ± 14.0 and 139.9 ± 26.41 μg/mg respectively) when compared to the diabetic control group (195.6 ± 48.51 μg/mg) with significant differences between the two groups.

The sitagliptin + denatonium benzoate combination group revealed a statistically significant decrease in UACR (56.25 ± 15.75 μg/mg) compared to diabetic control group and to the single drug groups.

#### Sitagliptin and denatonium-treated rats exhibited increased renal KLF6 expression

Sitagliptin group and denatonium group had a statistically significant increase in KLF6 expression (2.90 ± 0.81 and 1.81 ± 0.44 pg/ml respectively), as compared to the diabetic control group (0.80 ± 0.12 pg/ml) with significant difference between the two groups.

The sitagliptin + denatonium benzoate combination group exhibited a statistically significant increase in KLF6 expression (2.96 ± 0.86 pg/ml) when compared to the diabetic control and denatonium-treated group.

### Correlation results: Fig. 6

To reveal the role of KLF6 in diabetic renal complications in fructose-fed streptozotocin-induced diabetic rats, correlations were conducted between KLF6 expression in renal tissue and various parameters in all rats participating in the experiment. There were negative correlations between KLF6 expression with FBG (*r* = − 0.783), HbA1C% (*r* = − 0.808), LDL-C (*r* = − 0.796), TGs (*r* = − 0.855), and UACR (*r* = − 0.795). In addition, KLF6 expression was positively correlated with GLP (*r* = 0.781) and HDL-C (*r* = 0.701). All these correlations were statistically significant (*p* < 0.001).

**Fig. 6:**
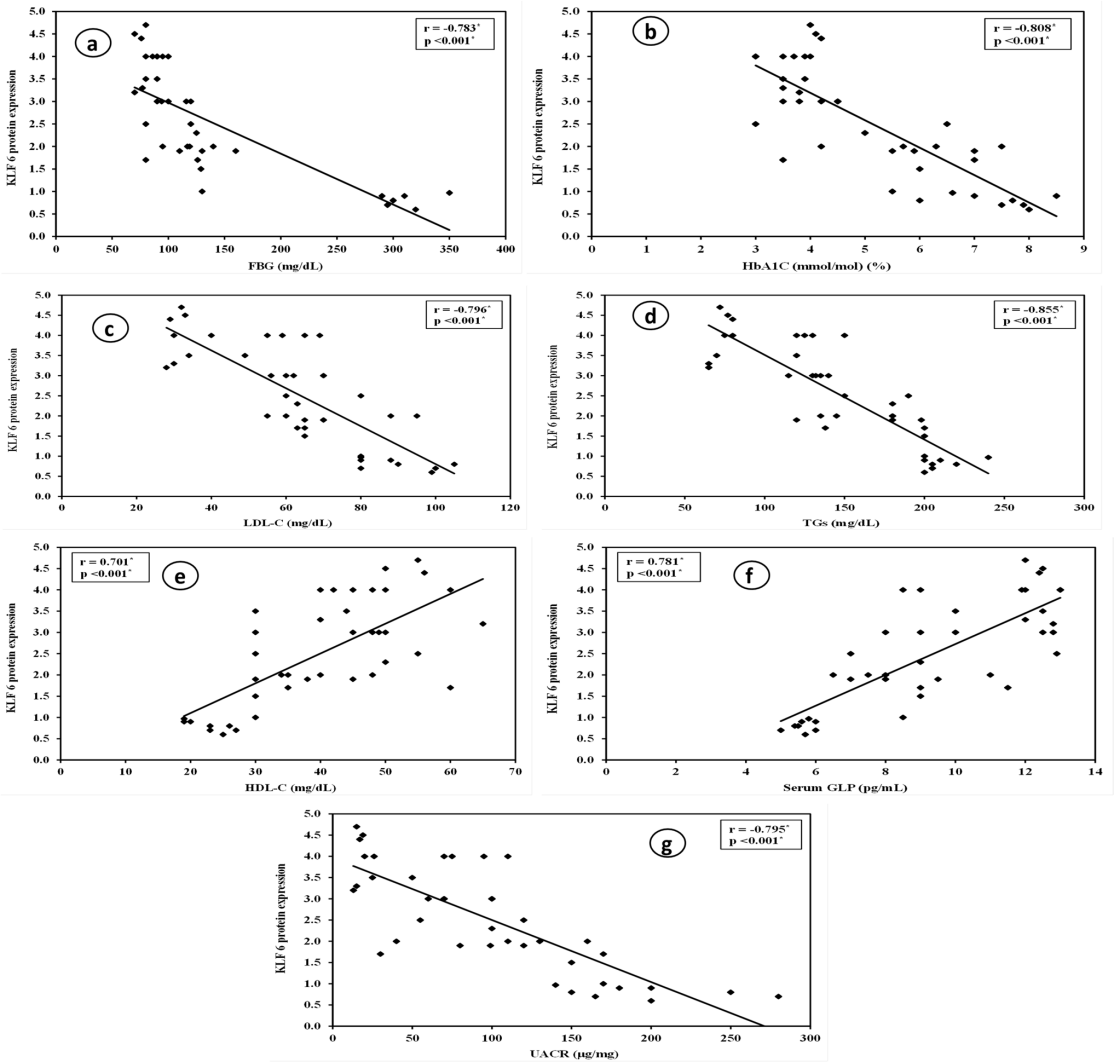
Pearson correlations between KLF6 expression with different studied biochemical parameters. **a** Negative correlation between KLF6 expression and FBG (mg/dl); **b** negative correlation between KLF6 expression and HbA1C (mmol/mol) (%); **c** negative correlation between KLF6 and LDL-C (mg/dl); **d** negative correlation between KLF6 expression and TGs (mg/dl); **e** positive correlation between KLF6 expression and HDL-C (mg/dl); **f** positive correlation between KLF6 expression and serum GLP (pg/ml); **g** negative correlation between KLF6 and UACR (μg/mg). *r*, Pearson coefficient; *statistically significant at *p* ≤ 0.05

### Histological examination: Fig. 7

The **normal control group** illustrated the renal cortex of normal architecture showing the glomerulus, lined by Bowman’s capsule, having an inner visceral layer lined by mesangial cells and podocytes separated by a urinary space and a well-developed outer parietal layer lined with simple squamous epithelium. The proximal convoluted tubules (PCT) were observed to have a narrow lumen and were lined with acidophilic cuboidal cells that displayed an apical brush border, while the distal convoluted tubule (DCT) showed a wider lumen and was lined by more cells at cross section.

**Fig. 7:**
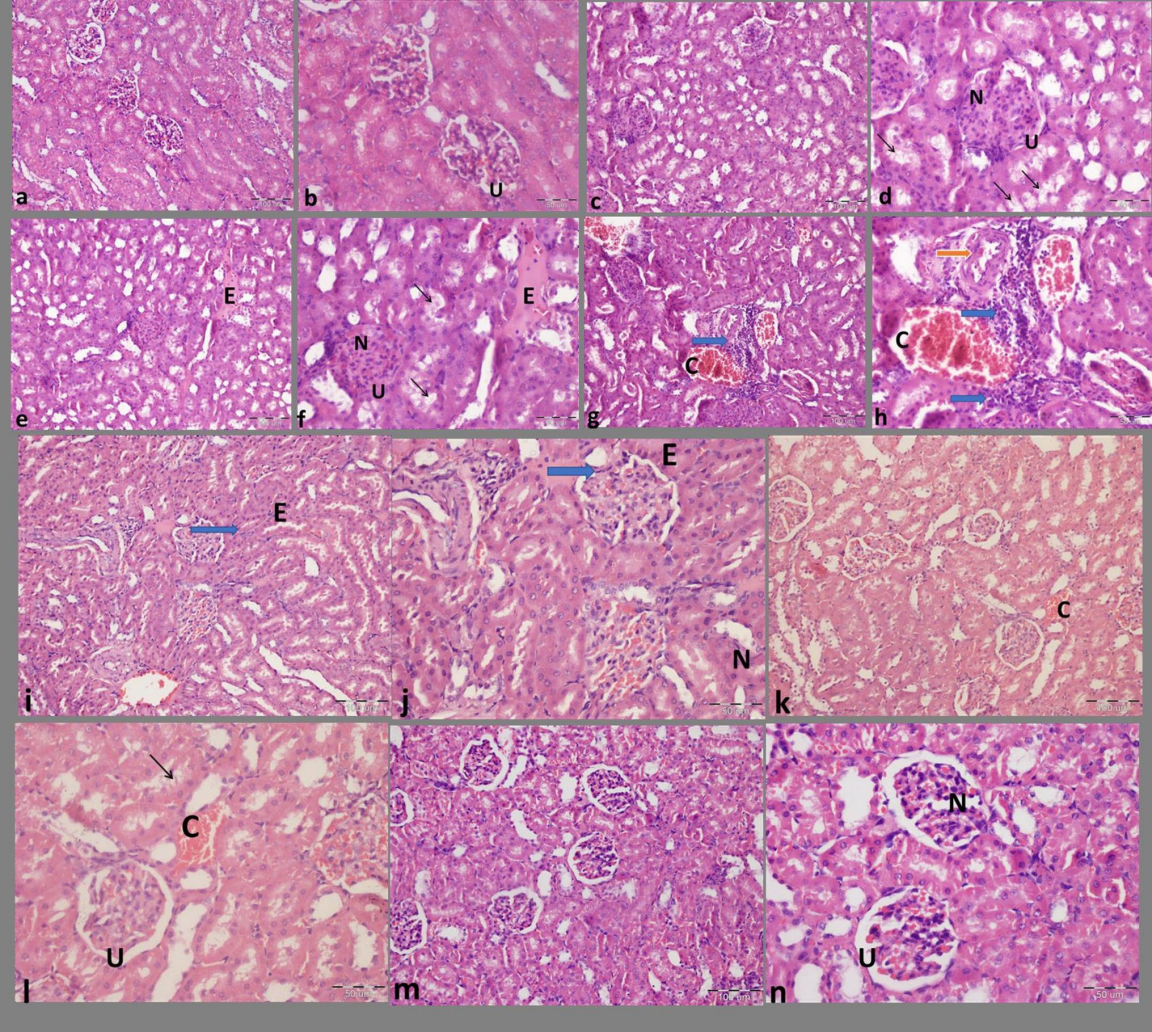
**a**, **b** Photomicrographs of rat kidney control group showing: The cortex shows renal corpuscles with normal Bowman’s capsule having well-developed outer parietal layer lined with simple squamous epithelium, and inner visceral layer lined by podocytes and mesangial cells separated by a urinary space (U). **c–h** Photomicrographs of rat kidney diabetic group showing: The cortex shows renal corpuscles with expanded glomeruli and narrowing or complete obliteration of the Bowman’s space (U). An increase in the number of the intra-corpuscular nuclei is noticed (N). Vacuolar degeneration in the surrounding tubules (black arrows) is noted. Note the presence of interstitial eosinophilic material (E). Interstitial inflammatory cells (blue arrows) are seen. Thickened arterial walls (red arrow) and congested blood vessels (C) are noticed. **i**, **j** Photomicrographs of rat kidney denatonium-treated group showing: The cortex shows renal corpuscle with mild decrease in the glomerulus size. There is an increase in the number of the intra-corpuscular nuclei (N). Note the presence of interstitial eosinophilic material (E) and inflammatory cells (blue arrow). **k**, **l** Photomicrographs of rat kidney sitagliptin-treated group showing: The cortex shows renal corpuscle with regained glomerulus size and urinary space (U). Vacuolar degeneration is seen in few of the surrounding tubules (black arrow). Note the presence of areas of congestion (C). **m**, **n** Photomicrographs of rat kidney sitagliptin and denatonium benzoate-treated group showing: The cortex shows normal renal corpuscles with preserved urinary space (U). The glomeruli appear with mild increase in the intra-corpuscular nuclei (N). (H&E stain, Mic. Mag. **a**, **c**, **e**, **g**, **i**, **k**, **m**: ×200; **b**, **d**, **f**, **h**, **j**, **l**, **n**: ×400)

On the other side, the **diabetic control group** illustrated a significant alteration in the glomerulus showing hypertrophy in its size with proliferation of the mesangial cells which appeared as an increase in the number of the nuclei. In addition, a decrease in the urinary space or even complete obliteration was evident. Many of the tubular cells show vacuolar degeneration. An interstitial eosinophilic deposition was seen. Congested blood vessels and thickened arterial walls were illustrated. Some inflammatory cells appeared in the interstitium.

The **sitagliptin-treated group** showed some signs of improvement in some glomeruli which regained their size and urinary space. But other glomeruli still showed increase in size and in intramesangial nuclei. Surrounding tubular vacuolization was still noticed. Also some areas showed congested blood vessels.

The **denatonium-treated group** revealed few signs of improvement in the cortical architecture. The glomeruli showed mild increase in size. Yet, the intramesangial nuclei showed increase in number in some glomeruli. Few areas in the interstitium showed eosinophilic deposition and inflammatory cells.

The **combined treatment group** with sitagliptin and denatonium showed the maximum improvement in architecture where the glomeruli appeared in normal size with preserved urinary space. There was a mild increase in the intra-corpuscular nuclei. Neither eosinophilic deposition nor inflammatory cells were seen.

### Histomorphometric results: Table 2 and Fig. 8

The diabetic control group illustrated statistically significant higher values of intracorpuscular nuclei count, mesangial expansion, and whole renal corpuscular volume, coupled with significantly lower values of Bowman’s space, than the normal control group. The kidney tissue damage scoring illustrated a normal control group of score 0, while the diabetic group was found to be score 3 according to all the parameters.

**Table 2:** Kidney tissue damage scoring

Group	Corpuscular volume score	Mesangial expansion score	Nuclei score	Bowman’s space score
Normal control	0	0	0	0
Diabetic control	3 (+83%)	3 (+71%)	3 (+350%)	3 (−63%)
Sitagliptin	1 (+33%)	1 (+29%)	2 (+175%)	1 (−22%)
Denatonium	2 (+58%)	2 (+36%)	3 (+250%)	2 (−38%)
Sitagliptin+Denat.	1 (+25%)	0 (≈0%)	2 (+100%)	0 (+9%)

**Fig. 8:**
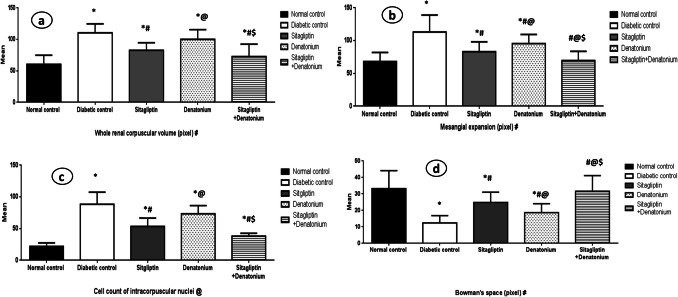
Effect of 8-week treatment with sitagliptin and denatonium alone or in combinations on whole renal corpuscular volume (pixel) in **a**, mesangial expansion (pixel) in **b**, cell count of intracorpuscular nuclei in **c**, and Bowman’s space (pixel) in **d**. Data are expressed as the mean±SD of eight rats per group. *p<0.05 significant vs. normal control, #*p*<0.05 significant vs. diabetic control, @*p*<0.05 significant vs. sitagliptin, $*p*<0.05 significant vs. denatonium. (*n* = 40). #30 replica in each group. @10 replica in each group

In comparison to diabetic control group, sitagliptin group had significantly lower values of intracorpuscular nuclei count, mesangial expansion, and whole renal corpuscular volume with significantly higher values of Bowman’s space. The kidney tissue damage score illustrated a score of 2 according to the nuclei count and a score of 1 according to the rest of the parameters.

Denatonium group displayed decreased values of intracorpuscular nuclei count and whole renal corpuscular volume which was insignificant, while this group showed significantly higher values of Bowman’s space and significantly lower mesangial expansion values than the diabetic control group. The kidney tissue damage score revealed a score of 3 according to the nuclei count and a score of 2 according to the rest of the parameters.

When compared to the diabetic control group and denatonium-treated group, the sitagliptin + denatonium benzoate combination group demonstrated a significant decrease in the numbers of intracorpuscular nuclei count, mesangial expansion, and whole renal corpuscular volume with significantly higher values of Bowman’s space. Kidney damage score ranged between 0 and 1.

## Discussion

One of the major unsolved consequences of diabetes mellitus that eventually leads to end-stage renal disease (ESRD) in people with both type 1 and type 2 diabetes is diabetic kidney disease (DKD) (El-kady et al.^2021^). It works by activating a number of metabolic, proinflammatory, and profibrotic pathways as a result of hyperglycemia and hyperlipidemia (Vallon and Komers 2011). Although current anti-diabetic medications have varying effects on the arms of these pathogenic pathways, controlling diabetic complications without side effects remains a challenge (Gerdes et al. 2023). This makes it necessary to look for alternative, different approaches to treatment. Our goal was to investigate the effects of denatonium benzoate, a bitter taste receptor agonist, both by itself and in conjunction with sitagliptin in diabetic rats.

In order to control the major risk factors for DKD, as hyperglycemia, hypertension, and dyslipidemia, pharmacotherapy is becoming more crucial with patient-specific treatments should be considered, taking into account parameters such as effect on body weight, level of HbA1C reduction, degree of chronic kidney disease, the presence of additional risk factors, safety and tolerability, and cost. Incretin-based therapies including the injectable GLP-1 receptor agonists (GLP-1RAs) and the orally active DPP-4 inhibitors are useful in management of patients with T2D, both of which stimulate insulin secretion and inhibit glucagon secretion in a glucose-dependent manner. GLP-1RAs have been shown to reduce HbA1C more than DPP-4 inhibitors and generate clinically meaningful weight loss in clinical trials, while DPP-4 inhibitors showed lesser glycaemic reductions and little effect on weight loss but both GLP-1RAs and DPP-4 inhibitors has low risk of hypoglycemia (Gilbert and Pratley 2020). As regarding the risk of major adverse cardiovascular events defined as a composite endpoint of stroke, myocardial infarction, and all-cause mortality, large randomized cardiovascular outcome trials have demonstrated that GLP-1RAs were associated with reduced risk of cardiovascular complications compared with DPP-4 inhibitors or sulfonylureas. At the same time, lower risk was observed with DPP-4 inhibitors when compared with sulfonylureas (Xie et al. 2023). 

**Fig. 9:**
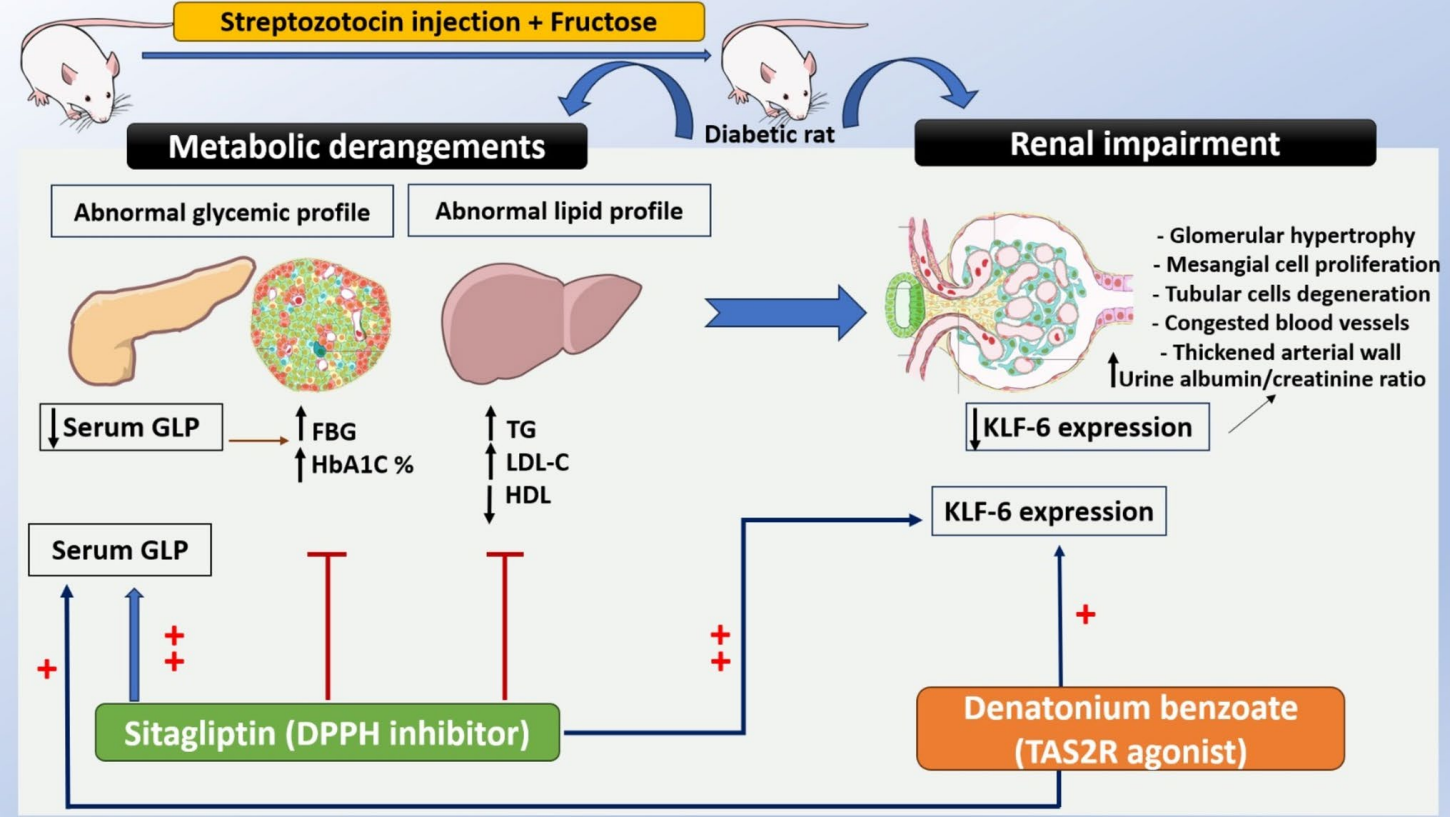
Diabetes induction with fructose-fed-STZ-induced rat model and the effects of treatment with sitagliptin and denatonium benzoate via modulation of GLP and KLF-6 expression. Images provided by the NIH BioArt source https://bioart.niaid.nih.gov/bioart/283, https://bioart.niaid.nih.gov/bioart/239, https://bioart.niaid.nih.gov/bioart/570, https://bioart.niaid.nih.gov/bioart/230, and https://bioart.niaid.nih.gov/bioart/562 were used in this chart

The present study employed the fructose-fed-STZ-induced rat model, which is one of the effective models to induce T2D in rats (Wilson and Islam 2012). Results of our present study revealed that the fructose-fed-STZ model simulated both metabolic and renal derangements observed in T2D. A statistically significant increase in FBG, HbA1C%, LDL-C, UACR, and TG levels and significant decrease in HDL-C, GLP level, and KLF6 expression were observed in diabetic control group as compared to normal control group (Fig. 9).

Microscopic examination of diabetic rates revealed an alteration in the glomerulus showing glucose-mediated hypertrophy and mesangial cell proliferation. The elevated glucose level that causes the early entry of mesangial cells from G0 to G1 phases may be the cause of this. However, following restricted proliferation, mesangial cells become growth-arrested in the G1 phase and do not advance into the S phase (Pourghasem et al. 2015). Furthermore, hyperglycemia in kidney cells can affect transforming growth factor-β (TGF-β) system which promotes cellular hypertrophy and stimulates the production of extracellular matrix (Ziyadeh et al. 2000). A decrease in the urinary space or even its complete obliteration was evident as a result of cellular proliferation and increased glomerular capacity. Many of the tubular cells showed vacuolar degeneration due to increased glycogen accumulation which occurs in severe hyperglycemia (Lau et al. 2013).

Additionally, an interstitial eosinophilic deposition was seen due to glycation of extra cellular matrix proteins (Sugimoto et al. 2007). Congested blood vessels and thickened arterial walls were also illustrated which is due to increased angiotensin 2 secretion in diabetes mellitus and is the most significant evidence of arterial hypertrophy and proliferation of smooth and mesangial cells (Gerdes et al. 2023).

KLF6 is an ubiquitous multifunctional member of the KLF family implicated in diabetes and diabetic complications. It serves as a vital protector of β-cell bulk and identity during insulin resistance and hyperglycemia conditions. It also limits dedifferentiation and transdifferentiation of β-cell into glucagon-producing α-cells (Dumayne et al. 2020) KLF6 also is a part of a network of interactions that control the glucose and lipid metabolism. It improves hepatic insulin resistance and regulates the posttranscriptional activation of peroxisome proliferator-activated receptor alpha (PPAR)-α is an chief nuclear receptor in the control of fatty acid oxidation that reduces total free fatty acid and triglyceride accumulation in macrophages (Bechmann et al. 2013). 

Moreover, previous work demonstrated upregulated KLF6 in periglomerular-activated fibroblasts during the progression of renal fibrosis, suggesting its conservative role in the development of renal tissue remodeling (Gallardo-Vara et al. 2016).

Our study documented the protective role of KLF6 through the negative correlation between KLF6 level and different studied parameters (FBG, HbA1C%, LDL-C, UACR, and TGs) and positive correlations between KLF6 and (HDL-C and GLP).

The current study looked at sitagliptin’s protective effects and found that all of the measures were statistically significantly improved. Both the histomorphometric data and the microscopic analysis of the renal cortices of diabetic rats given sitagliptin showed these effects.

Sitagliptin works by blocking the DPP-4 enzyme, which raises the amount of active GLP-1 in the body. By controlling glucose-stimulated insulin production and secretion from pancreatic β-cells and preventing glucagon release from pancreatic α-cells, GLP-1 has a beneficial effect on glucose homeostasis (Al-Qabbaa et al. 2023).

In addition to its strong insulinotropic effects, GLP-1 inhibits hunger, reduces stomach emptying, and promotes β-cell regeneration, all of which can enhance sitagliptin’s lipid-lowering effect (Shigematsu et al. 2014). Moreover, sitagliptin-mediated DPP4 inhibition could decrease the postprandial release of intestinal apoB-48-containing lipoproteins, hindering the postprandial accumulation of triglyceride-rich lipoprotein remnants, which play a major role in the development of atherosclerosis (Piccirillo et al. 2023).

Furthermore, DPP-4 is highly expressed in epithelial cells like renal glomeruli, where DPP-4 inhibitors exert their renoprotective effects. They can mitigate oxidative stress induced by high glucose (HG) in renal glomerular endothelial cells by inhibiting the synthesis of reactive oxygen species (ROS) such as Malondialdehyde (MDA) and the pro-inflammatory cytokines like interleukin-8 (IL-8) and interleukin-1β (IL-1β) (Haluzík et al. 2013).

Xu and Shao (2022) demonstrated that KLF6 mediated the suppressive effects of sitagliptin on the HG-induced inflammation, oxidative stress, and increased permeability in renal glomerular endothelial cells and reduction of the tight junction component claudin-5. Sitagliptin also stopped the decrease of KLF6 in human renal glomerular endothelial cells (HrGECs) caused by HG. Moreover, the renal protective effects of sitagliptin were eliminated by the silencing of KLF6 in HrGECs. This is in an accordance with our findings that revealed elevated levels of KLF6 expression in renal homogenate with sitagliptin-treated group.

On the other side, denatonium benzoate-treated group showed significantly lower levels of FBG, HbA1C%, TGs, LDL-C, and UACR with significantly higher HDL-C, GLP, and KLF6 expression than the diabetic control group. These results were evident by the microscopic analysis and the histomorphometric findings of renal cortices of diabetic rats given denatonium benzoate.

Reactions to bitter substances are mediated by TAS2Rs. Several investigations have detected the expression of these receptor proteins in numerous extra-oral tissues, despite the fact that they were first discovered in the apical microvilli of taste buds. In the gastrointestinal tract, they can modulate secretion of gut hormones like GLP-1, ghrelin, and Cholecystokinin (CCK), affect the motility of smooth muscle cells, insulin secretion, and innate immunological responses. Moreover, through peripheral effects on GIT motility and cerebral orexigenic signaling, activation of these receptors triggers the release of neurohormones, which in turn modulates food intake (Tong et al. 2025).

While about 25 human TAS2R genes are broadly expressed in taste buds of circumvallate papillae (type II taste cells), Behrens et al. (2007) found that renal TAS2R expression was found to be more selective as TAS2Rs were identified in the glomeruli, primary renal tubular epithelial cells, and the renal collecting tubule M-1 cells. The mouse kidney was also found to express α-gustducin, a key player in taste signal transduction (Liu et al. 2015). In the same context, in mice with diabetic nephropathy, activation of TAS2Rs reduced podocyte damage and improves renal functions (Gu et al. 2025). This indicates that TAS2Rs play an important role in maintaining the structure of the glomerulus and renal tubules. This can provide a basis for understanding the response of epithelial cells to the bitter compounds, which may be developed as pharmaceutical agents for the prevention and treatment of kidney disease (Liang et al. 2017). 

TAS2Rs are also overexpressed in adipocytes, where their stimulation by bitter agonists can help the delipidation of differentiated adipocytes and the prevention of lipid buildup during adipocyte differentiation. This shift in adipocyte metabolism might have contributed to support the positive effects of bitter taste agonists on glucose tolerance, insulin sensitivity, and lipid profile (Cancello et al. 2020).

The main mediator of denatonium benzoate’s antidiabetic benefits is the pleiotropic activities of GLP-1 signaling, which is modulated by bitter taste receptor signaling in the gut (Kok et al. 2018). Denatonium benzoate also showed anti-inflammatory and anti-hyperalgesic effects through PGE2 level reduction and provided protection against T2D-induced brain and testicular damage (Kokova et al. 2022), (Harby et al. 2025). In the same context, another study showed that denatonium benzoate stimulates insulin secretion through the closure of the K_ATP_ channel in pancreatic beta cells. It can also stimulate glucagon, somatostatin, and GLP-1 secretion from pancreatic islets, independent of glucose. On the other hand, the same study spotted that exposure to a high dose of denatonium benzoate induces cellular apoptosis in pancreatic islets which emphasize the importance of dose adjustment to gain the maximum metabolic benefits with the least toxic effects (Huang et al. 2024).

Together, these evidences demonstrate that TAS2Rs are chemosensory sentinels in a variety of organ systems, and their ability to alter hormonal, metabolic, food intake, and renal processes makes them attractive targets for future therapeutic activities.

At the same time, our present data revealed that denatonium benzoate group had a significant increase in KLF6 expression in the kidney, which should be highlighted as novel mechanism through which denatonium can perform its protective function in the kidney besides its metabolic and antidiabetic effects.

The best recorded outcomes were for sitagliptin + denatonium combination group with numbers close to normal control values and supported by the histological analysis. By contrasting this combination’s protective impact with that of each medication alone, its synergism may be determined. These encouraging findings should take a considerable interest that can be translated into a promising strategy for the management of diabetes and its renal complications. At the same time, more future studies are needed to validate and explain the possible mechanisms of the antidiabetic and renoprotective effects of denatonium benzoate and to determine the most effective therapeutic dose with minimal side effects.

## Conclusion

Our study suggests that KLF6 expression may play an important role in the pathophysiology of diabetes and its renal complications. The findings also indicate a potential metabolic and renoprotective effect of denatonium benzoate, either alone or in combination with sitagliptin, in fructose-fed streptozotocin-induced diabetic rats. These results highlight the need for further studies to clarify the underlying mechanisms and to evaluate their translational relevance in humans.

## Limitations

This study has some limitations. It was performed in a rat model of T2D, which may not fully reflect the complexity and heterogeneity of human diabetic nephropathy. Only a single dose and treatment duration of denatonium benzoate and sitagliptin were tested, and direct measures of renal function such as glomerular filtration rate were not included. Moreover, while changes in KLF6 expression were demonstrated, other relevant molecular pathways involved in TAS2R signaling and diabetic renal injury were not explored. Finally, the long-term safety and potential systemic effects of denatonium benzoate were not assessed, which warrants further investigation before clinical application can be considered.

## Supplementary Information

Below is the link to the electronic supplementary material.

Supplementary file1 (PDF 620 kb)

Supplementary file2 (DOCX 15 kb)

## Data Availability

The data that support the finding of the study are available on request from the corresponding author.
