# Supplementary material for: The possible renoprotective effect of denatonium benzoate in a rat model of type 2 diabetes: role of Krüppel-like factor 6 (KLF6)
Source: Naunyn Schmiedebergs Arch Pharmacol. 2025 Nov 5;399(4):5527–42. doi: 10.1007/s00210-025-04704-9 (PMC13046667; doi:10.1007/s00210-025-04704-9)
Supplement: Supplementary file 1 — Supplementary file1 (PDF 620 kb) [file 210_2025_4704_MOESM1_ESM.pdf]

Original western blots

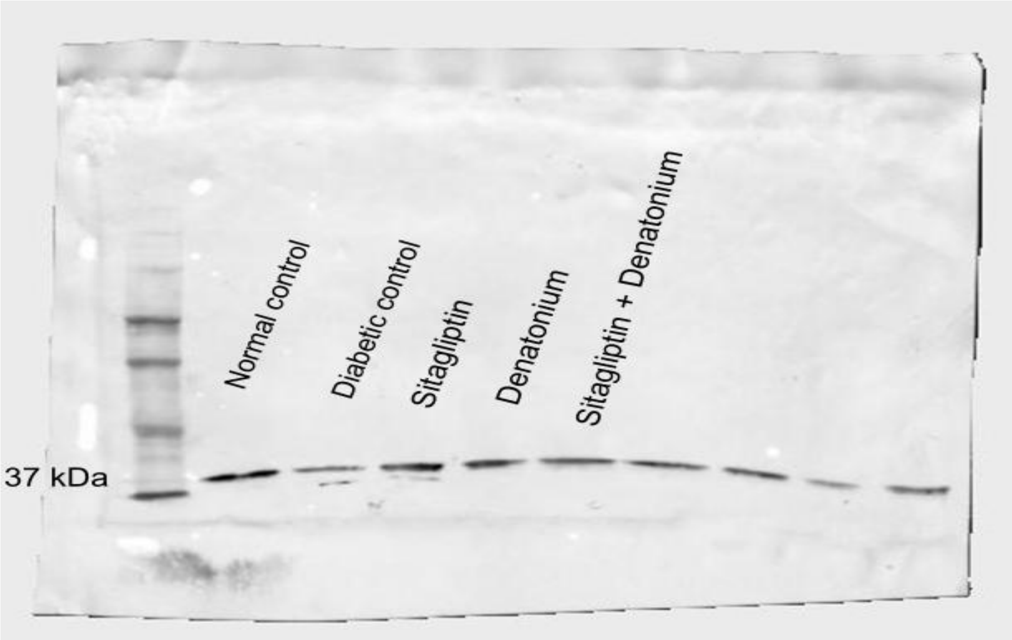	**KLF6**
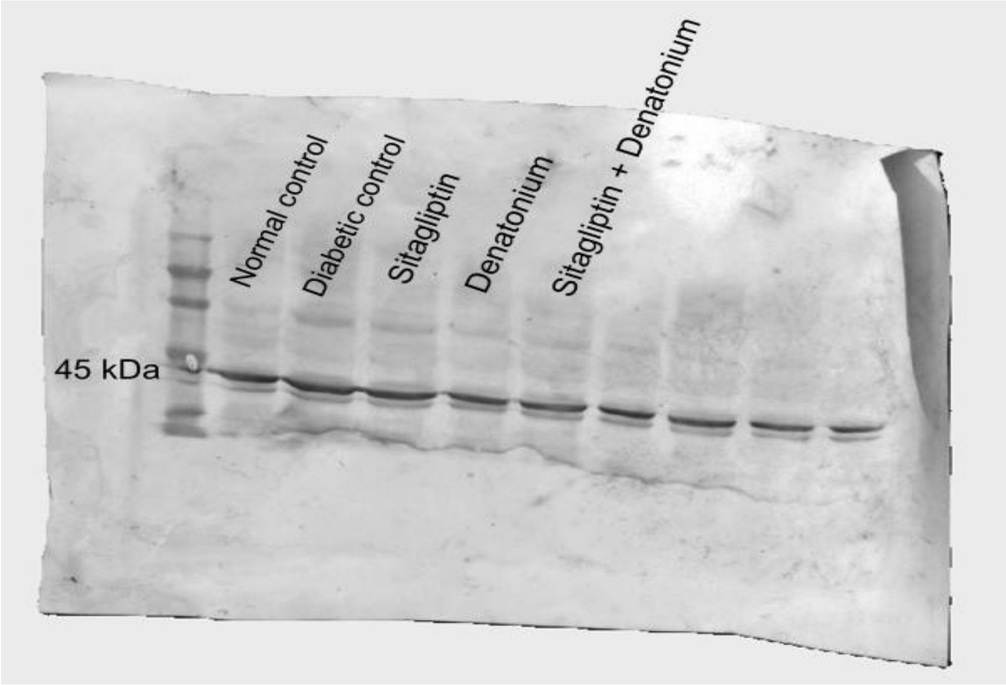	**β- actin**

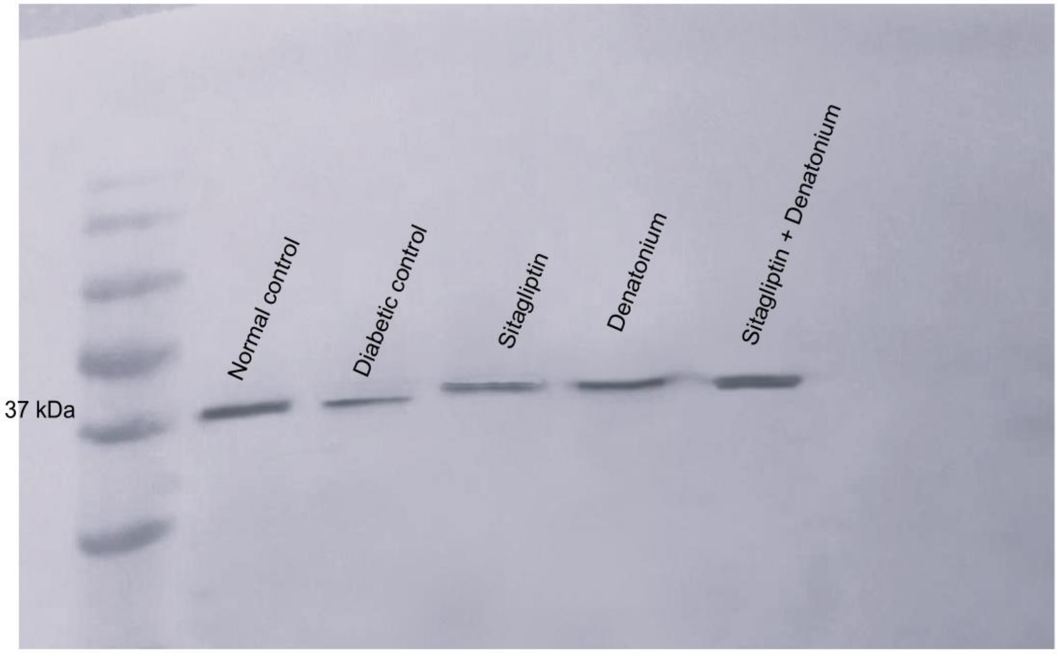 	**KLF6**
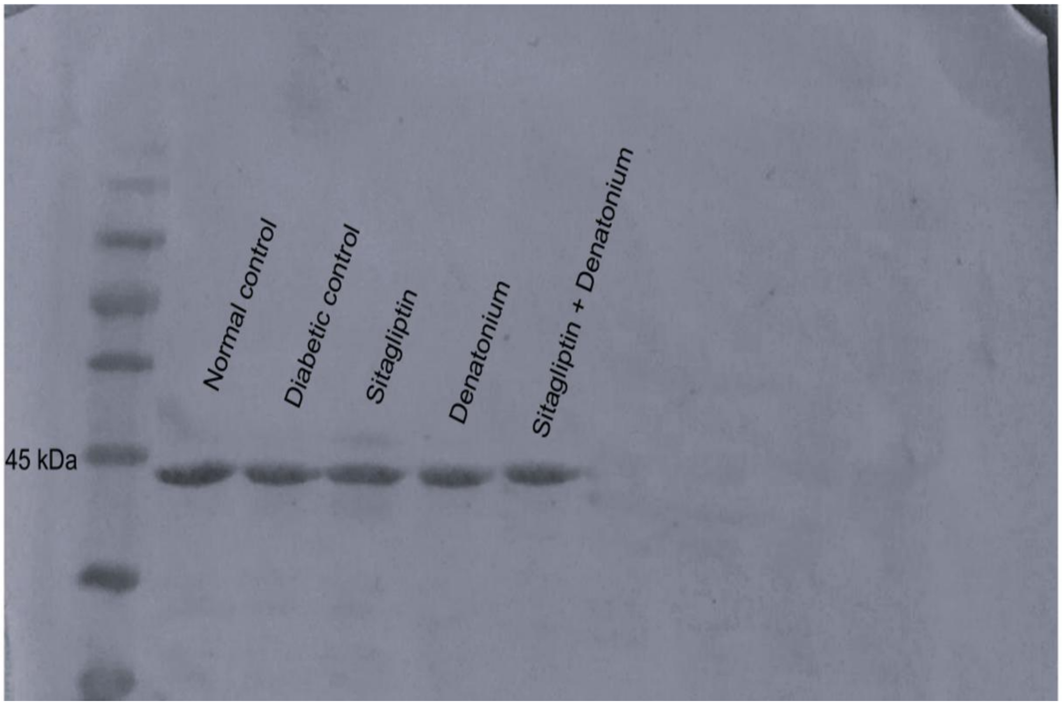 	**β-actin**

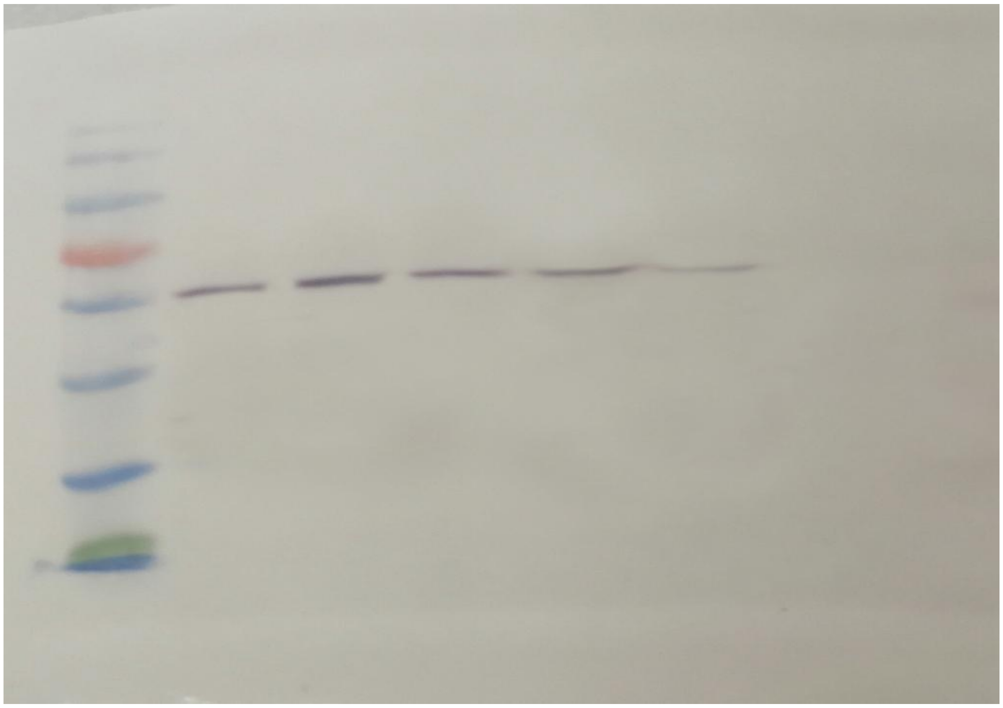	**KLF6**
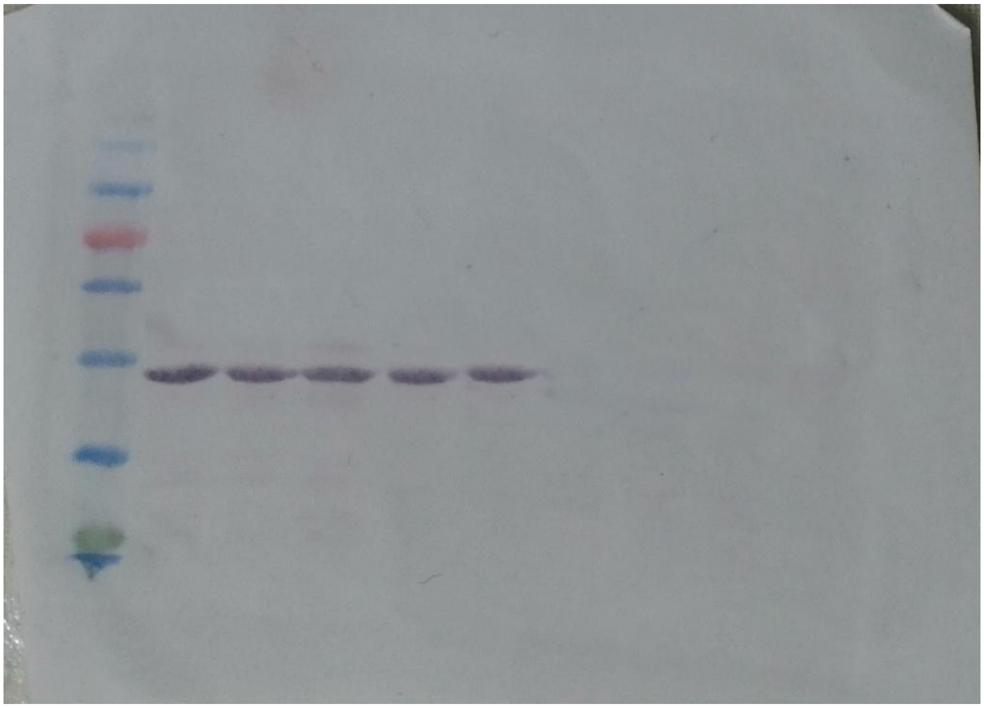	**β-actin**

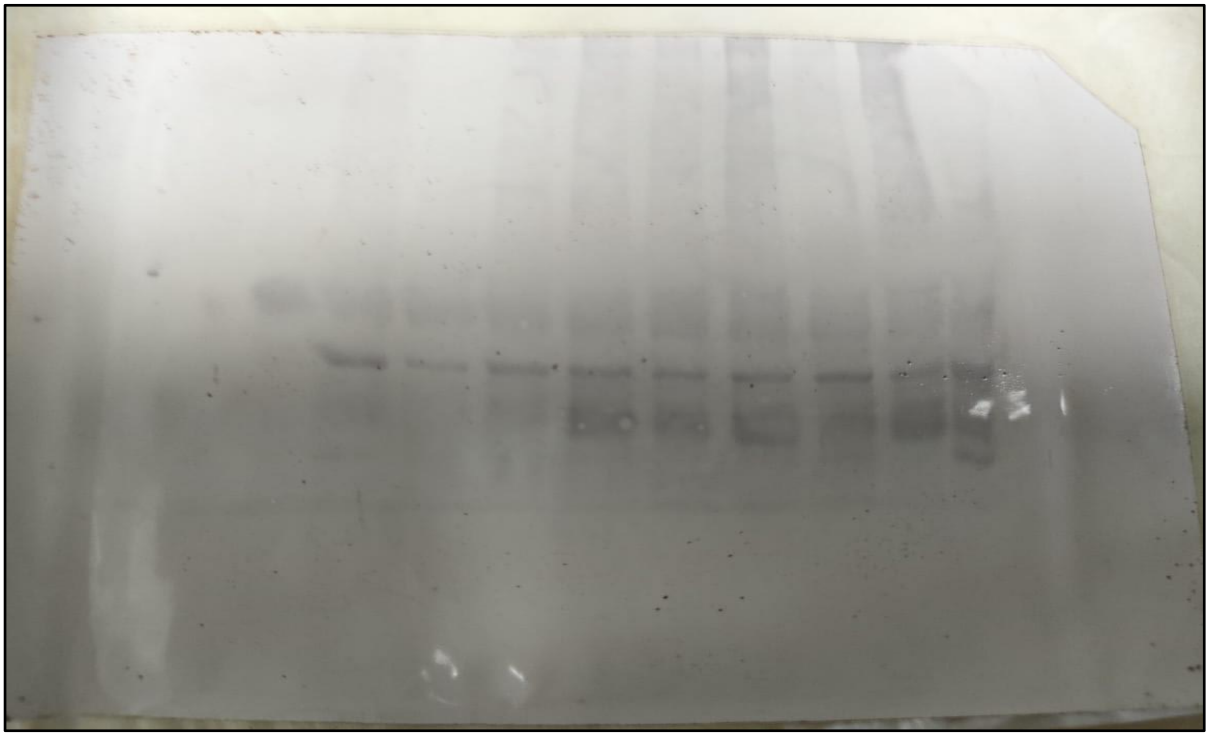

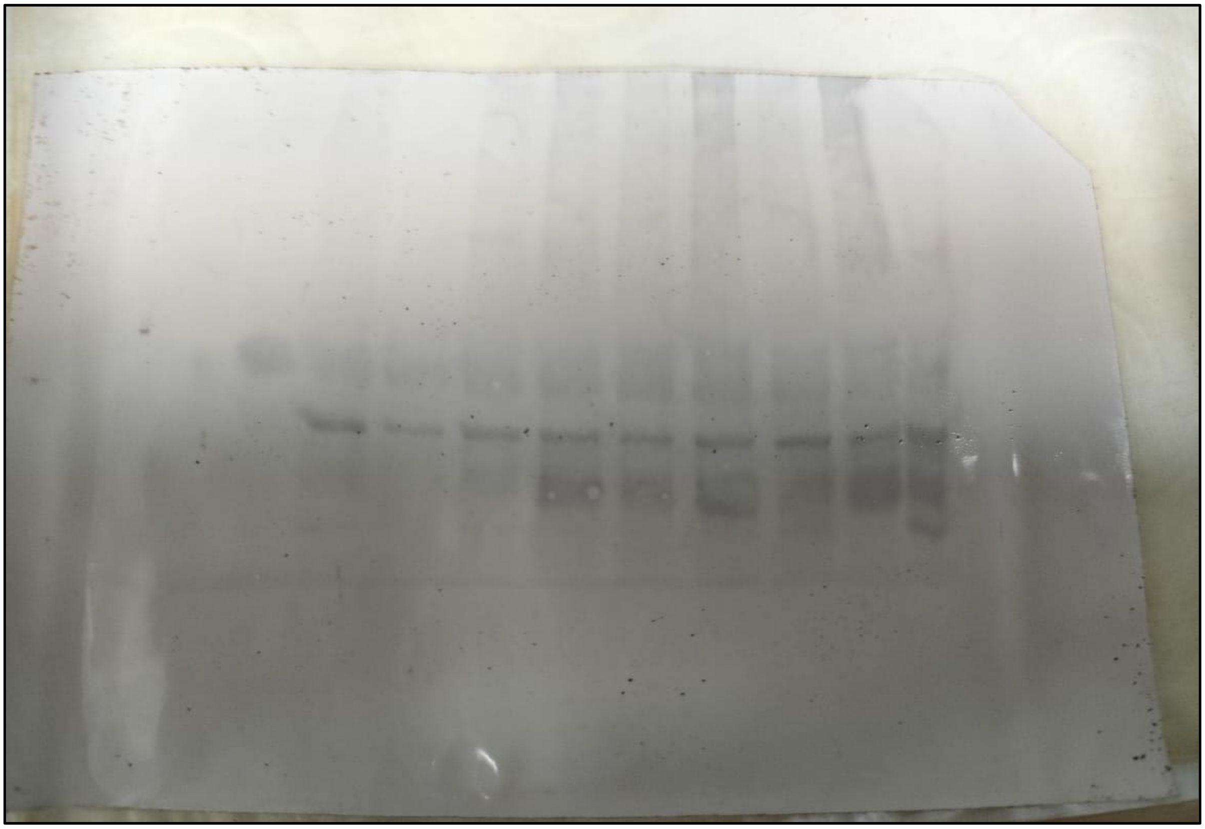

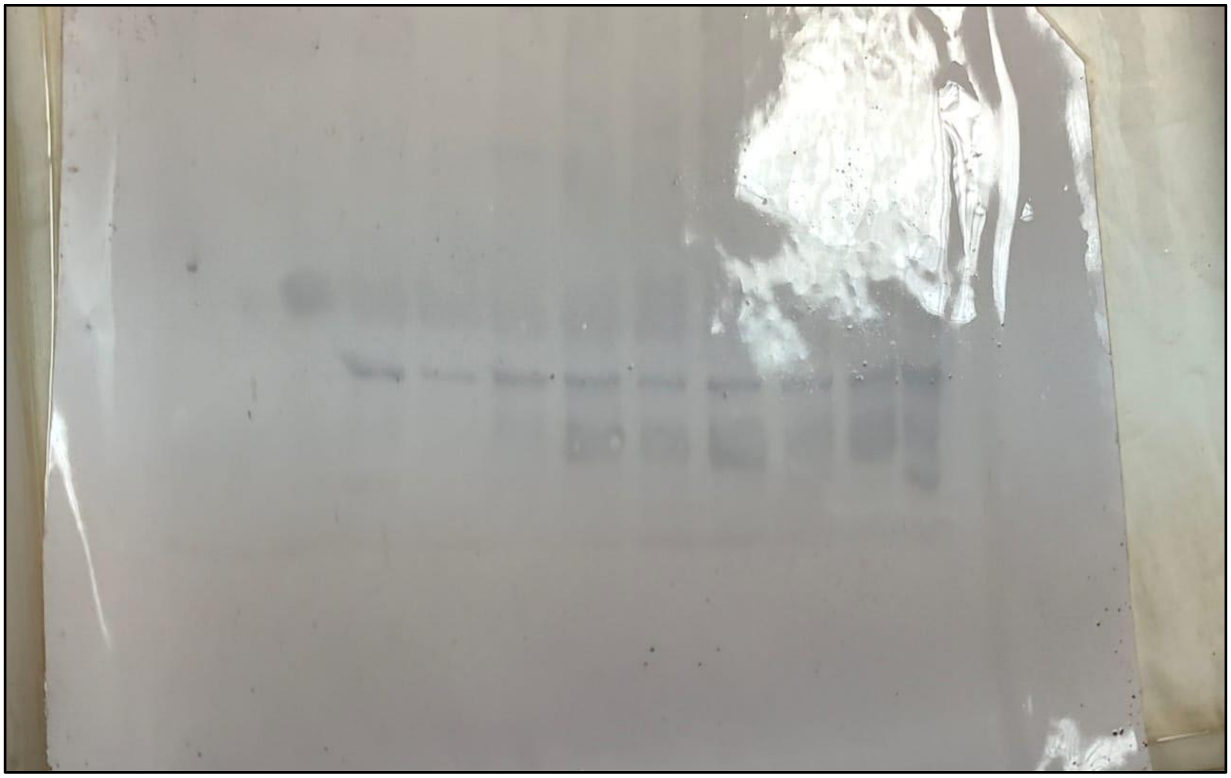

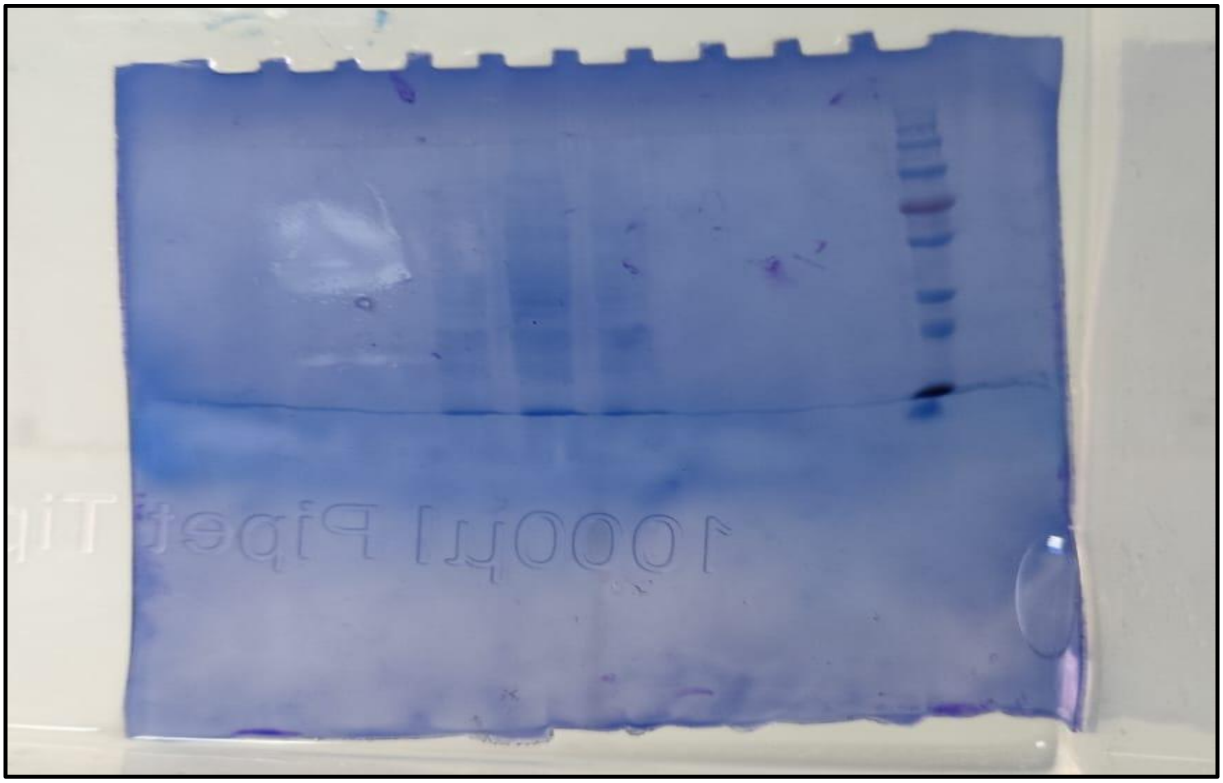

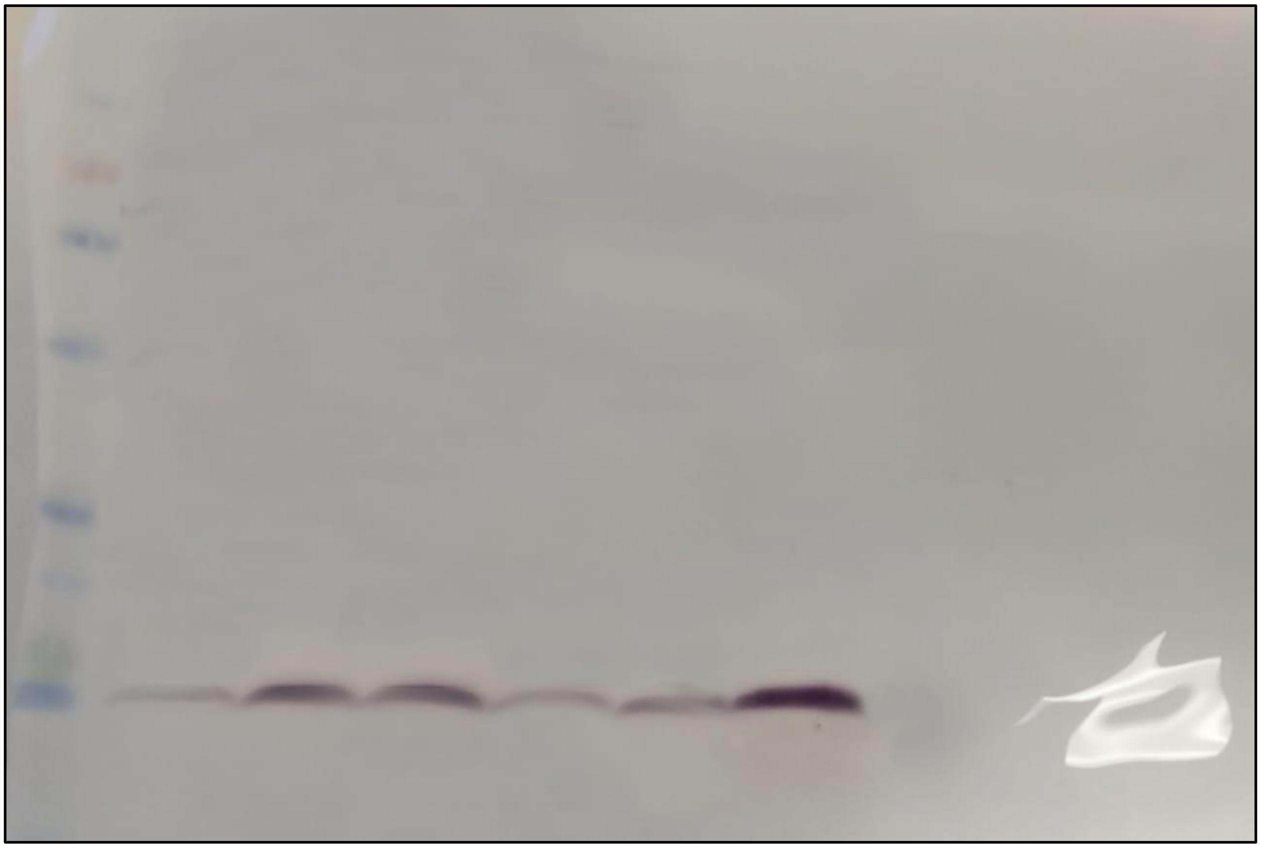

N.B:

The molecular weight of rat KLF6 protein is approximately 32 kDa based on the calculated molecular weight, and it is often observed at around 37 kDa in western blots due to post-translational modifications.
