# Supplementary material for: The possible renoprotective effect of denatonium benzoate in a rat model of type 2 diabetes: role of Krüppel-like factor 6 (KLF6)
Source: Naunyn Schmiedebergs Arch Pharmacol. 2025 Nov 5;399(4):5527–42. doi: 10.1007/s00210-025-04704-9 (PMC13046667; doi:10.1007/s00210-025-04704-9)
Supplement: Supplementary file 2 — Supplementary file2 (DOCX 15 kb) [file 210_2025_4704_MOESM2_ESM.docx]

**This table show the values of albumin for each group**

| **Sitagliptin+**  **Denatonium** | **Denatonium** | **Sitagliptin** | **Diabetic control** | **Normal control** | **Albumin (µg/mL)** |
| --- | --- | --- | --- | --- | --- |
| 686.00 | 1020 | 840 | 854 | 109.72 |  |
| 306.00 | 1120 | 850 | 795 | 140.25 |  |
| 380.00 | 720 | 900 | 1404 | 128.50 |  |
| 577.50 | 1224 | 790 | 907 | 161.00 |  |
| 700.00 | 845 | 890 | 1320 | 112.29 |  |
| 485.00 | 696 | 1064 | 1380 | 92.85 |  |
| 810.00 | 1139 | 800 | 1775 | 141.78 |  |
| 594.00 | 703 | 990 | 1596 | 238.16 |  |
| **567.31** | **933.4** | **890** | **1254** | **140.57** | **Mean** |
| **169.69** | **217.7** | **94.5** | **363** | **44.91** | **SD** |
